# Identification and characterization of novel and conserved microRNAs in several tissues of the Chinese rare minnow (*Gobiocypris rarus*) based on illumina deep sequencing technology

**DOI:** 10.1186/s12864-016-2606-5

**Published:** 2016-04-12

**Authors:** Xiangsheng Hong, Jianhui Qin, Rui Chen, Lilai Yuan, Jinmiao Zha, Zijian Wang

**Affiliations:** Key Laboratory of Drinking Water Science and Technology, Research Center for Eco-Environmental Sciences, Chinese Academy of Sciences, 18 Shuangqing Road, Haidian District, Beijing 100085 People’s Republic of China; Key Laboratory of Freshwater Animal Breeding, Ministry of Agriculture, College of Fisheries, Huazhong Agriculture University, Wuhan, 430070 China; Beijing Key Laboratory of Industrial Wastewater Treatment and Reuse, Research Center for Eco-Environmental Sciences, Chinese Academy of Sciences, Beijing, 100085 China; State Key Laboratory of Environmental Aquatic Chemistry, Research Center for Eco-Environmental Sciences, Chinese Academy of Sciences, Beijing, 100085 China

**Keywords:** *Gobiocypris rarus*, microRNA, Deep sequencing, Target prediction, IsomiRs

## Abstract

**Background:**

MicroRNAs (miRNAs), which comprise a large family of endogenous small non-coding RNA molecules, play important roles in the regulation of gene expression in various biological processes. The Chinese rare minnow (*Gobiocypris rarus*) is a Chinese native fish species and is used extensively as an experimental fish in China; however, relevant biological data, especially miRNA transcriptome data, have not been well documented. To discover conserved and potential novel miRNAs in Chinese rare minnows, a pool of equal amounts of RNA obtained from 6 different adult rare minnow tissues (brain, eye, gill, liver, muscle and heart) was sequenced using illumina deep sequencing technology.

**Results:**

In the present study, 26,930,553 raw reads, representing 2,118,439 unique high-quality reads, were obtained from the pooled small RNA library. Using bioinformatics analysis, 352 conserved and 112 novel Chinese rare minnow miRNAs were first discovered and characterized in this study. Moreover, we found extensive sequence variations (isomiRs) in rare minnow miRNAs, including internal miRNA isomiRs and terminal isomiRs at both the 5′ and 3′ ends and nucleotide variants. Six conserved and 4 novel miRNAs were selected and validated in 6 different adult rare minnow tissues using quantitative real-time PCR (qPCR). The results showed that miR-30a, miR-30b, and Novel-37 are ubiquitously expressed in a variety of tissues. miR-16a, miR-9, miR-125b, miR-34a, and Novel-69 were predominantly expressed in the brain. Novel-115 and Novel-7 were highly expressed in gills, but were relatively weakly expressed in other tissues. These results provided the expression patterns of miRNA genes in Chinese rare minnow. Finally, based on bioinformatics predictions, we mainly found that Novel-94 and Novel-1b-5p were simultaneously targeted to the 3′UTR of Dmrt1, which controls sex determination and/or sexual differentiation in a variety of metazoans at different sites. Novel-29b targeted the 3′UTR of Foxl2, which is involved in the maintenance of ovarian function and the transcriptional regulation of gonadal differentiation-related genes. Novel-62 and Novel-53 targeted the 3′UTR of ERbeta1 and ERbeta2 (which regulate the transcription of target genes), respectively.

**Conclusions:**

Rare minnow is a widely used model for assessing the risk of environmental pollution in China. Identifying and characterizing rare minnow miRNA genes is necessary to discover the biological function of miRNAs and to screen for new molecule biomarkers to assess the risk of environmental pollution in the future.

**Electronic supplementary material:**

The online version of this article (doi:10.1186/s12864-016-2606-5) contains supplementary material, which is available to authorized users.

## Background

MicroRNAs (miRNAs), a class of endogenous, approximately 22-nt-long, small regulatory RNAs [1], play important gene-regulatory roles in various biological processes [2, 3] including cell differentiation [4–6], proliferation [7], growth [7, 8], and aging and apoptosis [9, 10]. Since the first miRNA *lin-4* was characterized in *C. elegans* development [11], thousands of miRNAs have been found in animals and plants using genetic methods and through the sequencing of small RNA libraries [12, 13].

These small molecules can reduce the production of protein by modulating the stability and/or translational potential of their mRNA targets at the post-transcriptional level [1]. Generally, miRNAs are thought to function mainly by binding to target mRNAs through imperfect base paring with the 3′ untranslated regions (UTRs) and recruit the RNA-induced silencing complex (RISC), finally leading to down-regulation of their target genes [1, 14, 15]. Currently, a large number of miRNAs have been found in a variety of organisms from worms to humans, suggesting the evolutionary conservation of miRNA regulation mechanisms [1, 9, 11]. For instance, the let-7 miRNA gene, which was initially identified as a significant regulator that is involved in the heterochronic pathway controlling developmental timing in *C. elegans*, was the first miRNA known to be well-conserved from nematodes to primates [16, 17]. It is estimated that more than 60 % of mRNAs exhibit conserved miRNA-binding sites in mammals [18]. However, teleost miRNAs were first reported in zebrafish [14], and various miRNAs were found to play a role during zebrafish development; some of these functions have been characterized [19, 20]. Giraldez et al. [21] reported that miR-430 facilitates the deadenylation and clearance of maternal mRNAs during early zebrafish embryogenesis [22], and numerous studies have demonstrated the potential role of miRNAs in the regulation of tumorigenesis [23]. For example, miR-125b is an important negative regulator of p53 and p53-induced apoptosis during development and the stress response in zebrafish and humans [24]. Furthermore, through the combined use of miRNA microarray platforms and bioinformatics analysis, Craig et al. [25] identified changes in miRNA expression and related biological pathways in zebrafish following 7 days of exposure to fluoxetine (FLX). Furthermore, another study evaluated miRNAs in relation to toxin/chemical exposure in fish. Brzuzan et al. used qPCR to identify changes in the expression of 9 selected miRNAs in liver samples of whitefish following exposure to microcystin-LR (MC-LR) at a dose of 100 μg·kg^−1^ body weight for 24 or 48 h. Among these miRNAs, miR-122 exhibited the highest constitutive level, and this was associated with a decrease in the expression of genes coding for ferritin and a Ras-like protein [26].

The rare minnow (*Gobiocypris rarus*), a small freshwater cyprinid fish, is an endemic model organism that is mostly distributed in the upstream region of the Yangtze River, Sichuan Province, China. The fish are small (30–80 mm in length), are easy to culture in the laboratory, and have a relatively short life cycle, spawning hundreds of eggs with high fertilization and hatching rates [27, 28]. To date, this Cypriniformes species has been used extensively for various biological studies in China [29, 30]. Additionally, rare minnows have been widely applied in aquatic toxicity testing as a model vertebrate species for many years in our laboratory [27, 28, 30–34]. In the field of toxicology studies, several fish models are currently in use; however, research aimed at investigating miRNA alterations is mainly conducted in vitro, and in vivo studies remain in infancy [35]. The discovery of miRNA genes in the rare minnow genome will contribute to a better understanding of the roles played by miRNAs in regulating diverse biological processes in fish. Such studies are expected to prove useful for the future screening of novel molecule biomarkers to assess the risk of environmental pollution and in vivo toxicity.

## Methods

### Ethics statement

All fish were anesthetized with 2-phenoxyethanol before being euthanized. The fish were cared for in accordance with the Regulations for the Administration of Affairs Concerning Experimental Animals for the Science and Technology Bureau of China throughout the study.

### Fish

The rare minnow has been maintained in our laboratory for over 13 years. The brood stock was maintained in a flow-through system based on dechlorinated tap water and was subjected to a 16:8 h light:dark cycle at 25 ± 1 °C [27]. The brood stock was fed with newly hatched brine shrimp (Artemia nauplii) twice daily and granulated food (TetraMin, Tetra Werke, Melle, Germany) once daily. In this study, ten healthy adult rare minnows (5 months old) were selected for study. The average body weight and body length were 1.2 ± 0.4 g and 42.3 ± 3.7 mm, respectively.

### Sample collection

Tissue samples including the brain, eye, gill, liver, muscle and heart were collected from adult rare minnows. All samples were immersed in liquid nitrogen immediately after collection and stored at −80 °C. Total RNA was isolated from the tissue samples using a mirVana™ microRNA Isolation Kit (Ambion, USA), according to the manufacturer’s instructions. RNA integrity was examined using the Agilent 2100 Bioanalyzer system (Santa Clara, CA, USA); the samples were then stored at −80 °C until analysis.

### Small RNA library construction and sequencing

In this study, one miRNA library was constructed, and an equal quantity (10 μg) of total RNA was extracted from each of the various tissue samples. Briefly, 15–35 nt molecules were purified from total RNA (mirVana™ microRNA Isolation Kit, Ambion, USA) using polyacrylamide gel electrophoresis (PAGE); then, 5′ and 3′ adaptors (TruSeq® Small RNA Sample Preparation Kit, Illumina, USA) were ligated to the 5′ and 3′ termini of the RNAs, and the samples were used as templates for cDNA synthesis. Subsequently, PCR amplification was performed using primers that were complementary to both adaptors. After purification of the amplified cDNA constructs, the products were sequenced using HiSeq2500 technology. All sequencing reads were deposited in the National Center for Biotechnology Short Read Archive (SRA) database (http://www.ncbi.nlm.nih.gov/sra/) under the study accession SRP057175.

### Bioinformatics analysis

The original reads obtained from small RNA sequencing were processed by summarizing data production, evaluating sequencing quality and calculating the length distribution of the small RNA reads. The quality assessment and processing of sequenced reads were performed as recommended [36–38]. Briefly, the quality of the sequenced reads was assessed using the FastQC application (FastQC-0.11.2, http://www.bioinformatics.bbsrc.ac.uk/projects/fastqc/). Adapter sequences from the reads were removed with FASTX-toolkit (FASTX-toolkit 0.0.14, http://hannonlab.cshl.edu/fastx_toolkit/commandline.html). After adaptor trimming, filtering low quality tags and removing reads either < 18 nt or > 30 nt, the remaining reads were mapped to the zebrafish genome with a tolerance of one mismatch in the seed sequence. Subsequently, using BLAST against the Rfam (http://Rfam.sanger.ac.uk/) database, the reads that were mapped to the zebrafish genome were analyzed to annotate rRNA, tRNA, snRNA, snoRNA and other ncRNA sequences in the small sequences. Conserved rare minnow miRNAs in the sequences were then identified using BLAST searching against the miRNA database, miRBase (release 21, http://www.mirbase.org/) and no more than two mismatches can be allowed to identify homologs of known miRNAs. The sequences that did not match known miRNAs were mapped to the Ensembl (http://www.ensembl.org) zebrafish genome (Zv9) and rare minnow EST and GSS databases to identify potentially novel miRNA candidates; these were identified by folding the flanking genome sequence of unique small RNAs using MIREAP (https://sourceforge.net/projects/mireap/).

### Validation of miRNAs by qPCR analysis

qPCR was used to validate six conserved and four novel selected miRNAs, and the relative expression levels were analyzed in six tissues from adult rare minnows, including the brain, liver, heart, muscle, eye and gill. Total RNA was isolated using the miRcute miRNA Isolation Kit (TIANGEN, Beijing, China) according to the manufacturer’s instructions. Then, two micrograms of total RNA from each sample were reverse-transcribed into cDNA using the miRcute miRNA First-Strand cDNA Synthesis Kit (TIANGEN, Beijing, China). After this was completed, qPCR was performed using the miRcute miRNA qPCR Detection Kit (TIANGEN, Beijing, China). The following reaction solution was prepared on ice: 10 μL 2 × miRcute miRNA Premix (containing SYBR & ROX), 0.4 μL PCR forward primer (10 μM), 0.4 μL Uni-miR qPCR Primer (10 μM, part of the Kit), 1.6 μL cDNA, and RNase-free dH_2_O up to 20 μL. The reaction mixtures were incubated in a 96-well plate at 94 °C for 2 min followed by 40 cycles of 94 °C for 20 s and 60 °C for 34 s. All primers used in the reverse transcription and qPCR experiments are shown in Additional file 1: Table S1. Samples (*n* = 3) were run simultaneously for each gene in triplicate, and a non-template control was included in each plate. The relative miRNA expression level was calculated using the 2^−ΔΔCt^ method after the threshold cycle (Ct); the 5S rRNA (submitted to the European Nucleotide Archive, http://www.ebi.ac.uk/ena/data/view/LN878980) was used as an endogenous control. miRNA expression levels are presented as 2^−ΔΔCt^ mean ± SE (standard error), and error bars indicate the standard error of 2^−ΔΔCt^ mean values.

### Identification of Novel miRNA targets via computational analysis

Target prediction was performed by integrating four miRNA target prediction software packages, including miRanda (http://www.microrna.org/microrna/home.do) [39], PITA (http://genie.weizmann.ac.il/pubs/mir07/mir07_exe.html) [40], RNA22 (https://cm.jefferson.edu/rna22/) [41] and TargetScan (http://www.targetscan.org/fish_62/) [42]. To obtain a better miRNA target analysis result, we used the local version of these four software packages. All newly identified miRNA sequences were used to query the ten gene sequences of rare minnows using the default parameters.

## Results and discussion

### Illumina sequencing of small RNAs

**Fig. 1:**
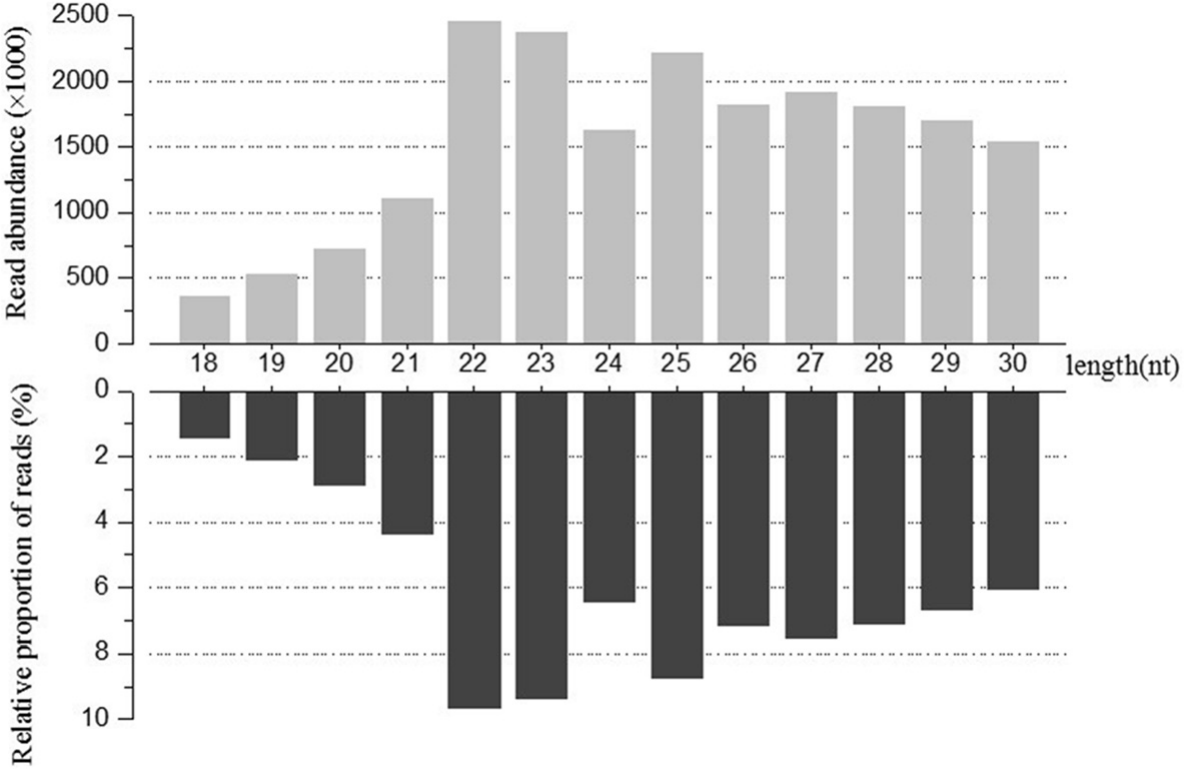
Length distribution of small RNA sequences. Sequence length distribution of total reads based on abundance; the most abundant size class was 22 nt, followed by 23 nt and 25 nt

To increase the coverage of rare minnow miRNAs, a small RNA library was constructed from the pooled RNA samples that were collected from six adult rare minnow tissues and sequenced; The total of 26,930,553 raw reads were generated by deep sequencing using illumina deep sequencing technology. After low-quality sequences, adaptors and contaminated sequences were eliminated, 25,434,468 (94.44 %) high quality reads of longer than 18 nt remained. Small RNA sequences between 18 and 30 nt (Fig. 1) were selected for further analysis; such sequences accounted for 74.84 % of the total raw reads. Examination of the reads after such a grouping revealed a main size class, among which most reads were 22 and 23 nt; this finding is consistent with RNA sequence results obtained for channel catfish [43] and zebrafish [44]. A length distribution analysis showed that 36.1 % of the reads were between 18 and 24 nt. After comparing the small RNA sequences with the Rfam database, rRNA, tRNA, and snoRNA and other ncRNA sequence reads were annotated (Table 1); these sequences accounted for 26.94, 1.48 and 0.03 % of the total sequences, respectively. Because whole genome data for the rare minnow are not available, we aligned the selected small RNA sequences to the Ensembl (http://www.ensembl.org) zebrafish genome (Zv9) sequence (zebrafish is the evolutionarily most closely related species with an available sequenced genome) to perform a distribution analysis at the genomic scale. Among the selected reads, 15,956,842 sequences from the small RNA library mapped to the zebrafish genome in our screen for candidate miRNA sites, accounting for 62.74 % of all reads; 732,306 reads were obtained representing conserved miRNAs in Metazoa.

**Table 1:** Read statistics of the obtained small RNAs

Items	Total reads	Unique reads
Raw reads	26,930,553	
High-quality reads (> =18 nt)	25,434,468	2,118,439
Aligned to the zebrafish genome	15,956,842	160,650
Conserved miRNAs in Metazoa	732,306	2,222
Non-conserved in Metazoa	15,224,536	158,428
Reads for novel miRNA prediction	7,709,152	53,473
rRNA	6,851,768	33,644
tRNA	375,439	8,725
snoRNA	6,984	1,261
Other	185,255	8,400

In summary, the total number of sequence reads generated by high-throughput sequencing using the Illumina platform was of the same order of magnitude (10^7^) as previous results for zebrafish [44]; however, this number was significantly different (10^6^) from that obtained for Nile tilapia [45]. These differences are due to the evolutionary relationships between the species; zebrafish and rare minnows are both cyprinids, whereas Nile tilapia belongs to cichlidae.

### Conserved and Novel miRNAs in rare minnow

To identify conserved miRNAs in rare minnows, perfectly matched reads from the small RNA library were searched against Metazoa mature miRNAs in miRBase (Release 21). Alignment of the sequences to the miRBase database revealed that 732,306 of all sequences yielded a positive match to known miRNAs. Finally, we identified 352 conserved miRNAs (Additional file 2: Table S2) in rare minnow.

Illumina small-RNA deep sequencing enabled us to determine species-specific miRNAs. The sequences were compared with the zebrafish genome sequence to detect potential novel miRNA candidates when the sequences did not match known miRNA precursors. Moreover, precursors with two or more unique reads that were located at mature positions were deemed of high probability. In this study, 112 putative novel miRNA/miRNA* candidates were identified that matched the zebrafish genome and rare monnow EST and GSS databases with a tolerance of two mismatches. RNA stem-loop structures and chromosome positions of these candidates are shown in Additional file 3: Table S3 and Additional file 4: Table S4, respectively. As shown in Table 2, isomiR species of the novel miRNA candidates are quite different. For instance, Novel-117 represented just three types of isomiRs, whereas Novel-40a represented more than 100 isomiRs.

**Table 2:** The 10 most frequent highly expressed novel miRNA candidates

Name	Total count	isomiRs	Most expressed sequence	Length(nt)	Counts	Seed
Novel-21	553	31	UUACCACAGGGAUAACUG	18	232	UACCACA
Novel-16	270	4	AUCAUUUUUGUGACUAUGCAACU	23	264	UCAUUUU
Novel-7	668	9	UGAGAACUGAAUUCCAAGGGUGU	23	332	GAGAACU
Novel-127	3,127	28	AGGUGUUGGUUGAUAUAGACA	21	389	GGUGUUG
Novel-117	744	3	CUGACGUGCAAAUCGGUC	18	484	UGACGUG
Novel-118	5,064	63	UGCGGACCAGGGGAAUCC	18	484	GCGGACC
Novel-115	3,368	25	CGAAUGACUAGAGGCCUUGG	20	526	GAAUGAC
Novel-40b	1,879	4	UUGACUCUAGUCUGGCAC	18	1096	UGACUCU
Novel-59	13,127	78	CUGGCACUGUGAAGAGACAUGAGG	24	1204	UGGCACU
Novel-51	9,024	51	UGCGGGAUGAACCGAACGCC	21	1234	GCGGGAU
Novel-50	1,738	4	AACGGGCUUGGCAGAAUC	18	1272	ACGGGCU
Novel-33	3,867	15	GAUUAUGACUGAACGCCU	18	1540	AUUAUGA
Novel-15b	8,773	80	AUGGCGCUGGAGCGUCGGGCCC	22	1693	UGGCGCU
Novel-15a	16,536	59	GAUGGCGCUGGAGCGUCGGGCCC	23	2001	AUGGCGC
Novel-13	7,224	19	CAGGAUAGCUGGCGCUCGCC	20	2087	AGGAUAG
Novel-40a	117,202	112	UUGACUCUAGUCUGGCACUGUGAAGA	26	6095	UGACUCU

Abundant miRNAs play essential and broad regulatory function in biological processes. In the current study, the most highly expressed miRNA in rare minnows was miR-215 (51,777 reads). Previous studies have indicated that miR-215 is involved in a variety of cancers in mammals. For example, Deng et al. found that miR-215 is significantly up-regulated in gastric cancer tissues from either gastrectomy or gastroscopy by targeting retinoblastoma tumor-suppressor gene 1 (RB1) through its 3′-UTR in gastric cancer cells [46]. Moreover, White et al. demonstrated that miR-215 overexpression decreased cellular migration and invasion in a renal cell carcinoma (RCC) cell line model using miRNA microarray and qPCR analyses [47].

### Multiple isomiRs in the rare minnow

**Fig. 2:**
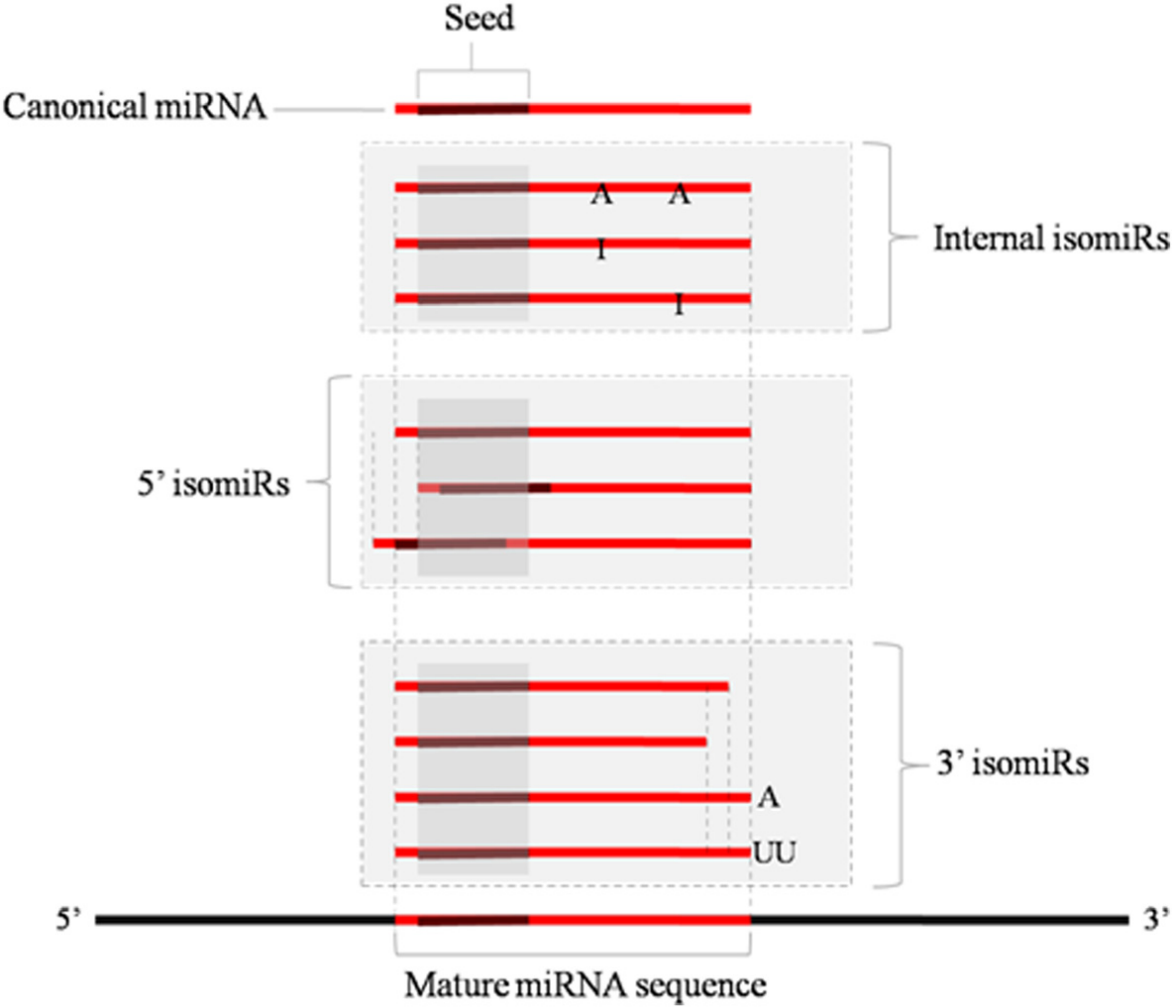
Schematic representation of microRNA isomiR species

**Fig. 3:**
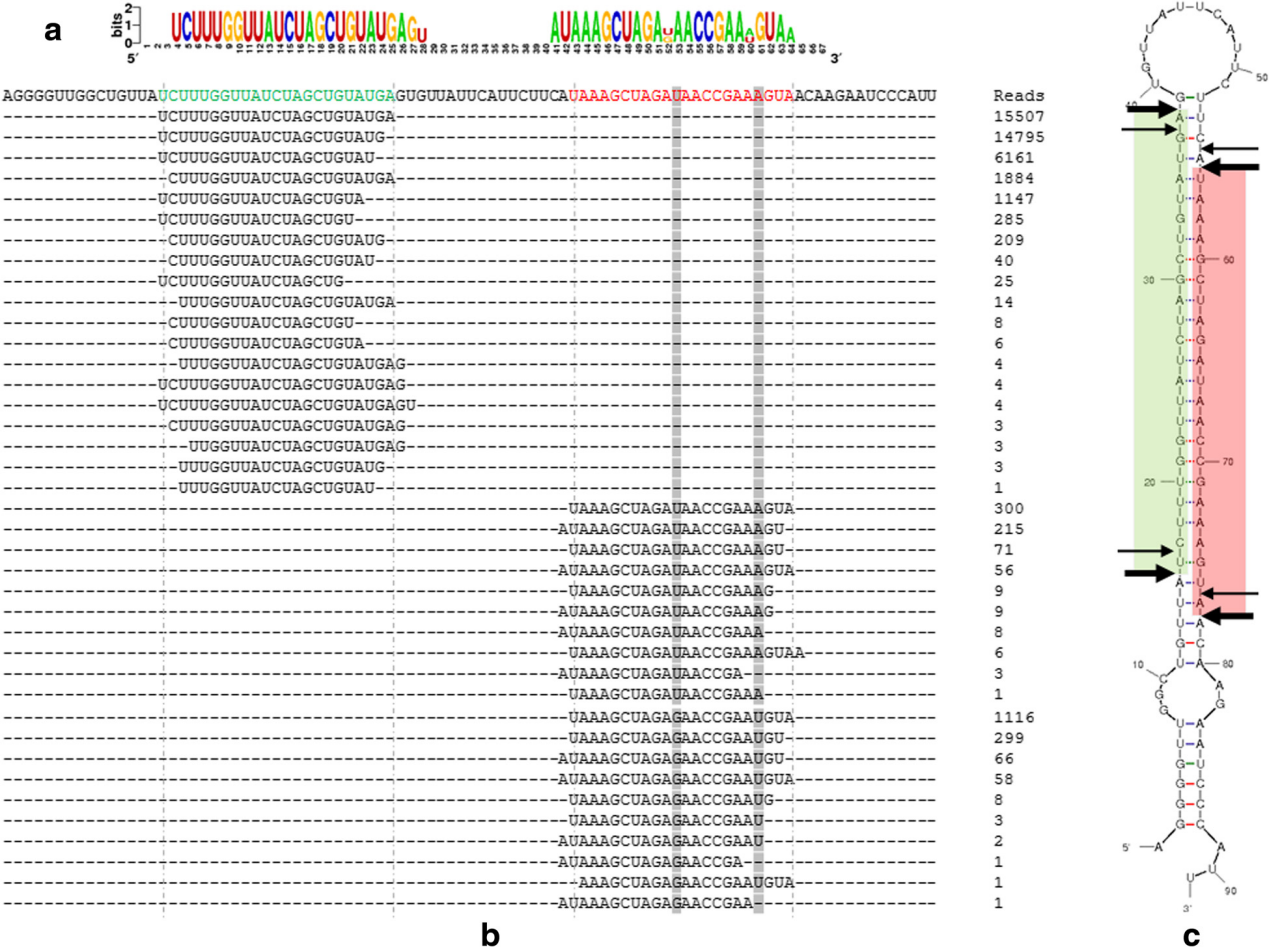
miR-9 sequence heterogeneity. **a** Significant sequence variation was detected among mature miRNA reads. The figure shows a compilation of miR-9 variants; the extent of variation at any particular nucleotide is indicated by the size of the font by WebLogo [74]. **b** (*Below*) The mature 5p miRNA sequence is shown in *green*, and the 3p miRNA sequence is shown in *red*; the adjacent genomic sequence is shown in *black*. Single nucleotide substitutions are highlighted in *gray*. The number of unique reads that are aligned to the putative precursor is listed on the right side of the figure. **c** A graphic illustration of the hairpin-loop precursor is shown on the right of the figure. The dominant cleavage sites are indicated using black arrows. *Large arrows* show the most abundant isomer cleavage sites, whereas *small arrows* indicate less abundant isomiR cleavage sites. The most abundant mature miRNAs (5′p and 3′p) are shown by the regions shown in *green* and *red*

IsomiRs are physiological miRNA variants that can be classified as 5′, internal and 3′ isomiRs [48], rather than the experimental artifacts derived from RNA degradation during sample preparation for next-generation sequencing (NGS) strategies [49, 50]. In our study, all three types of isomiRs (Fig. 2) were widely found. For example, in our Solexa library, almost all rare minnow miRNAs frequently exhibited variation from their reference sequences, and multiple mature variants occurred (hereafter referred to as isomiRs). The most abundant miRNA in the brain, miR-9 is illustrated in Fig. 3. In total, 39 isomiRs (19 from 5p, 20 from 3p) were confirmed as miR-9. Among all isomiRs, the predominant type was 3′ isomiRs (53.66 %). Interestingly, trimming of the 3′ end of the miRNAs accounted for 99.96 % of the 3′ isomiRs. Initially, most isomiRs, particularly those exhibiting 3′ variability, appear to be functionally redundant. However, previous studies have reported that isomiRs affect miRNA targeting efficiency, AGO incorporation, and stability [50–52]. 3′ uridylation promotes isomiR degradation, whereas 3′ adenylation increases the stability of isomiRs in plants [53, 54]. However, the effect of 3′ uridylation and adenylation on the stability of mature miRNAs is not well documented in animals. In this study, we found that trimming of the 5′ end of miRNAs and internal isomiRs (located in the out-seed position) accounted for 4.48 and 3.67 %, respectively. Although generally rarer than 3′ isomiRs, 5′ isomiRs (e.g., miR-9, less than 10 %) are predicted to strongly affect mRNA targeting. Because the 5′ end of a miRNA defines its seed sequence, a single nucleotide shift at this site will radically alter its target selection [55, 56]. In vitro target RNA cleavage assays have demonstrated that miR-142 and its 5′ isomiR (+5′ CC) exhibit different targeting effects on a fully complementary artificial reporter [57]. In another study, luciferase assays revealed that CDH1 with 3′UTRs matching the seed region of canonical miR-9 were not obviously depressed by isomiRs (−5′U), whereas the 5′ isomiR (−5′ U) of miR-9 gained the ability to significantly inhibit the expression of DNMT3B and NCAM2 [58]. Our results revealed that the isomiRs were also abundant in Rare Minnow and further studies were warranted to validate their roles.

### Validation and analysis of miRNA expression

**Fig. 4:**
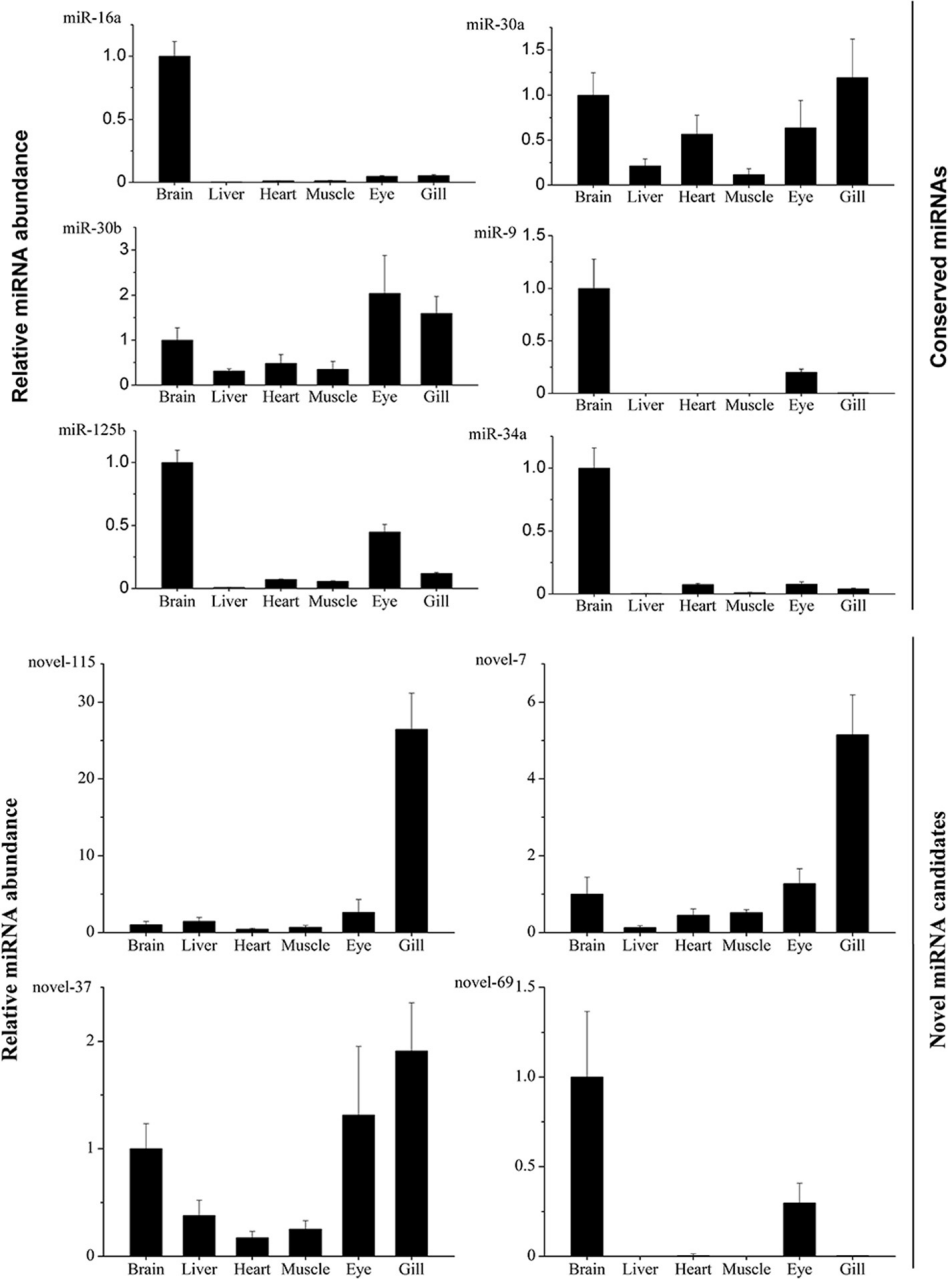
Tissue distribution of the miRNAs analyzed using qPCR. Tissue type is shown on the X axis. The relative miRNA expression level is shown on the Y axis. The results are presented as fold changes; rare minnow 5S rRNA is used as an endogenous control, and the fold change is expressed relative to the expression in brain. Values are shown as mean ± standard deviation (*n* = 3)

Our sequencing data were validated using the oligo (dT) primer method [59]. Ten miRNAs, including 6 conserved miRNAs and 4 novel miRNAs, were selected and validated in rare minnow tissues (brain, liver, heart, muscle, eye and gill) using qPCR. The distribution of miRNAs was evaluated using 5S rRNA as an endogenous control. As shown in Fig. 4, some miRNAs, such as miR-30a and miR-30b, were expressed in all tissues examined. The ubiquitous nature of this expression suggested that these miRNAs might be associated with fundamental processes, such as metabolism [60–62]. On the other hand, some miRNAs exhibited highly differential patterns of tissue distribution. Novel-7 and Novel-115 were prominently expressed in the gill, whereas miR-125b, miR-34a, miR-16a, miR-9, and Novel-69 were mainly expressed in the brain (Fig. 4). Among them, the expressions of miR-16a, miR-125b and miR-34a were consistent with previous findings [12, 19, 63]. The results obtained for miR-9, another important miRNA that exhibits brain-specific expression in fish and mammals [15, 64], are consistent with our results. Moreover, Leucht et al. demonstrated that miR-9 can co-regulate the organizing activity and progenitor state of the midbrain-hindbrain boundary (MHB) during late embryonic development in zebrafish [64]. Our results showed the tissue specific expression of miRNAs in Chinese rare minnow, including conserved miRNAs and novel miRNA candidates (Fig. 4). However, the function of these miRNAs remains to be understood in the future study.

### Identification of Novel miRNA targets via computational analysis

**Fig. 5:**
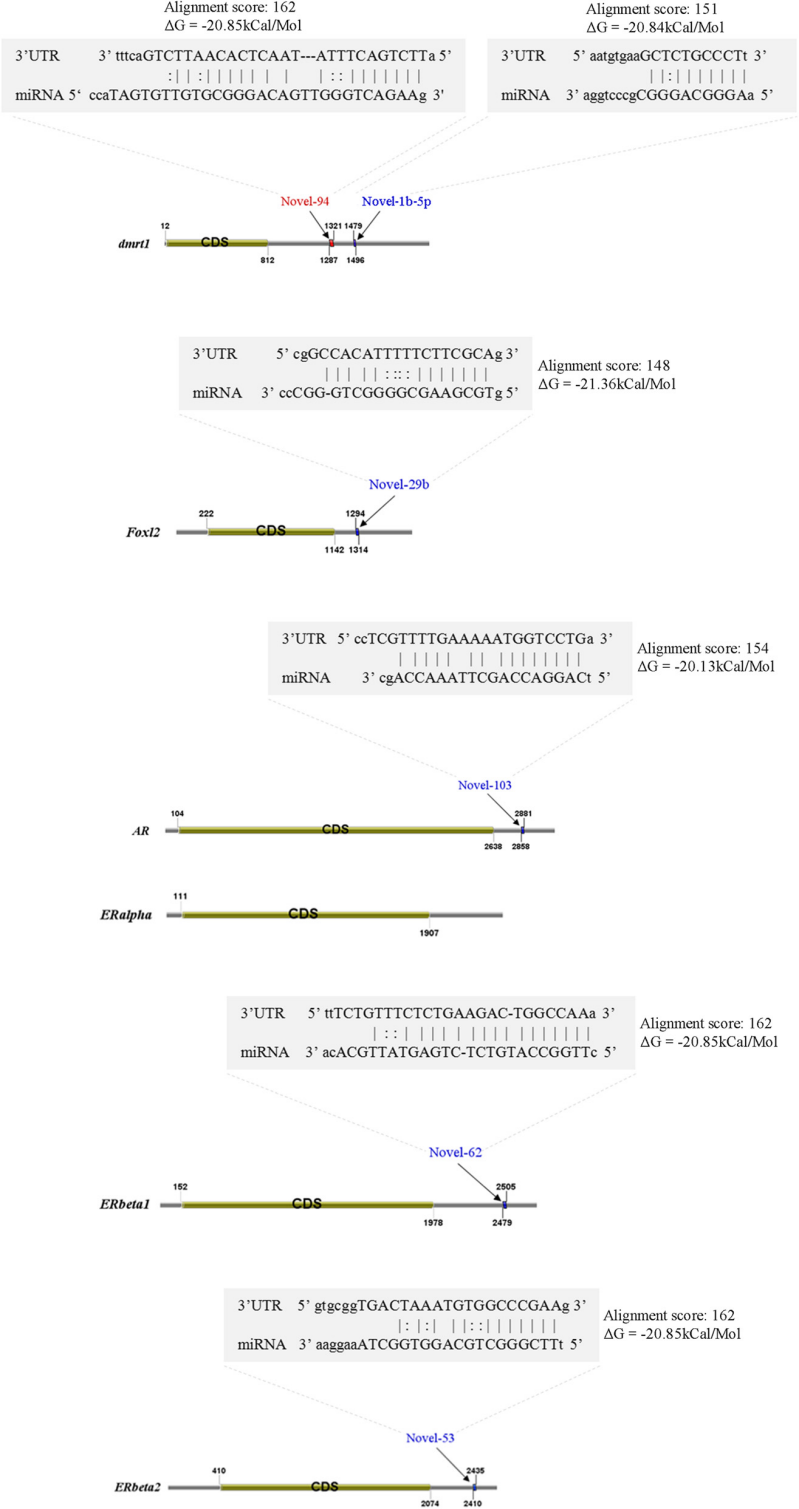
A schematic illustration of miRNA and its putative binding sites in the 3′UTR of selected genes. Novel miRNAs target 6 rare minnow genes using a 3′UTR binding site; these genes include *dmrt1*, *Foxl2*, *AR*, *ERalpha*, *ERbeta1* and *ERbeta2*, according to predictions made using miRanda, TargetScan, RNA22 and PITA (a local version). The obtained predictions were identical among the four algorithms. The miRNA:mRNA sequence alignments, together with the calculated score and the minimum free energy of the duplex, are presented above each target binding site. In the alignment, “|” refers to perfect complementary between bases, “-” represents a gap, and “:” represents a G:U wobble pair

To further explore the potential biological function of novel miRNAs in the rare minnow, we integrated four algorithms including miRanda, TargetScan, RNA22 and PITA to predict putative miRNAs that target ten selected rare minnow genes (Additional file 5: Table S5). The predictions were conducted using a local version of the software due to the absence of rare minnow miRNAs in current versions of the above mentioned algorithms. Using miRNA target prediction, we obtained the putative binding site of Dmrt1 based on its 3′UTR targeting by the potential novel miRNAs Novel-94 and Novel-1b-5p (Fig. 5). Previous studies have shown that Dmrt1 controls sex determination and/or sexual differentiation in a variety of metazoans [63, 65]. Recently, Lindeman et al. reported that loss of the Dmrt1 transcription factor in adult mice can cause the transdifferentiation of testicular Sertoli cells to ovarian granulosa cells. Interestingly, the ectopic expression of Dmrt1 can reprogram granulosa cells to Sertoli-like cells in the ovary [66]. Our findings might aid in studies of the sex-determination mechanism. In addition, one of the putative novel miRNAs, Novel-29b, might target the 3′UTR of forkhead box L2 (Foxl2). Foxl2, a transcription factor, is mainly involved in the maintenance of ovarian function and in the transcriptional regulation of gonadal differentiation-related genes in fish [67–70]. Our results showed that Novel-29b might be related to the action of Foxl2 by targeting its 3′UTR. In addition, Novel-62 and Novel-53 were considered likely to target the 3′UTR of ERbeta1 and ERbeta2, respectively. Estrogen receptors (ERs, which are ligand-inducible nuclear receptors) regulate the transcription of target genes such as genes involved in cell cycle control, including proto-oncogenes and cyclin genes [71–73]. Our findings indicated that Novel-62 and Novel-53 might be involved in regulating the estrogen receptor in the rare minnow. These results suggest a number of general clues regarding potential miRNA-mRNA regulatory relationships. However, these regulatory relationships require further investigation. Therefore, further studies regarding the function of these novel miRNAs are warranted; such studies might improve our understanding of the role of miRNAs in the mechanism of sex differentiation.

## Conclusion

This work represents an initial study on the miRNA profile of the Chinese rare minnow. The differential expression of miRNAs and the prediction of their target genes provide a basis for further understanding rare minnow miRNAs and the biological processes in which they are involved. The rare minnow is a model species for various biological studies in lower vertebrates in China. The identification and functional characterization of rare minnow miRNAs reported here will provide new opportunities for functional genome research on the rare minnow and should prove useful when screening for novel molecule biomarkers for use in assessing the risk of environmental pollution.

## Additional files

Additional file 1: Table S1. Primer sequences used in the qPCR assays. (XLSX 12 kb) Additional file 2: Table S2. Conserved miRNAs and miRNA*s in rare minnow. (XLSX 26 kb) Additional file 3: Table S3. Putative novel microRNAs with sizes between 18 and 30 nt. (XLSX 1321 kb) Additional file 4: Table S4. The location of putative novel microRNAs on the zebrafish whole-genome shotgun (WGS). (XLSX 30 kb) Additional file 5: Table S5. Novel miRNA target prediction using miRanda, TargetScan, RNA22 and PITA (local version). (XLSX 515 kb)
